# UVB Radiation Delays *Tribolium castaneum* Metamorphosis by Influencing Ecdysteroid Metabolism

**DOI:** 10.1371/journal.pone.0151831

**Published:** 2016-03-17

**Authors:** Wen Sang, Lin Yu, Li He, Wei-Hua Ma, Zhi-Hui Zhu, Fen Zhu, Xiao-Ping Wang, Chao-Liang Lei

**Affiliations:** 1 Hubei Insect Resources Utilization and Sustainable Pest Management Key Laboratory, Huazhong Agricultural University, Wuhan, China; 2 Key Laboratory of Plant Pathology of Hubei Province, Huazhong Agricultural University, Wuhan, China; University of Cincinnati, UNITED STATES

## Abstract

Ultraviolet B (UVB) radiation is an important environmental factor. It is generally known that UVB exhibits high genotoxicity due to causing DNA damage, potentially leading to skin carcinogenesis and aging in mammals. However, little is known about the effects of UVB on the development and metamorphosis of insects, which are the most abundant terrestrial animals. In the present study, we performed dose-response analyses of the effects UVB irradiation on *Tribolium castaneum* metamorphosis, assessed the function of the *T*. *castaneum* prothoracicotropic hormone gene (*Trcptth*), and analyzed ecdysteroid pathway gene expression profile and ecdysterone titers post-UVB irradiation. The results showed that UVB not only caused death of *T*. *castaneum* larvae, but also delayed larval-pupal metamorphosis and reduced the size and emergence rate of pupae. In addition, we verified the function of *Trcptth*, which is responsible for regulating metamorphosis. It was also found that the expression profiles of *Trcptth* as well as ecdysteroidogenesis and response genes were influenced by UVB radiation. Therefore, a disturbance pulse of ecdysteroid may be involved in delaying development under exposure to irradiation. To our knowledge, this is the first report indicating that UVB can influence the metamorphosis of insects. This study will contribute to a better understanding of the impact of UVB on signaling mechanisms in insect metamorphosis.

## Introduction

Solar ultraviolet radiation (UVR) is an important environmental factor in ecosystem and is classified into three categories: UVA (315–400 nm), UVB (280–315 nm), and UVC (200–280 nm) [1]. UVC is rarely reported to play a role in ecology due to its complete absorption by the ozone layer. Although UVA constitutes approximately 95% of solar UV radiation reaching the earth’s surface, it is regarded as a far less harmful environmental stressor because of its low DNA damaging ability. UVB constitutes only 5% of solar UV radiation, but it exhibits high genotoxicity due to the formation of cyclobutane pyrimidine dimers and (6–4) photoproducts through the excitation of nucleobases and oxygen-independent reactions, causing mutations. Thus, UVB has received more attention [2–4]. A considerable amount of research has mainly focused on detrimental effects on mammals, such as skin carcinogenesis and aging [5–7]. However, it is currently unclear how UVB radiation affects other important biological processes, such as development and metamorphosis, in other organisms. A few studies have referred to the effects of UVB on amphibians, such as *Rana clamitans* Latreille, *Rana aurora* Baird and Girard, and *Rana pipiens* Schreber, which include arrest and delay of the development and metamorphosis of larvae and reduction of larval mass [8–10]. However, little is known about the effects of UVB on the development and metamorphosis of insects, which are the most abundant group of terrestrial animals [11, 12].

The metamorphosis of holometabolous insects is a biological process through which immature larvae develop into last-instar larvae, undergo holometabolism through the inactive pupal stage and finally emerge as mature adults capable of reproduction and flying. It is one of most widely observed life-history strategies exploited by insects to find new food resources, disperse, and multiply. A complex series of programs leads to total reorganization of the body plan during metamorphosis, which includes larval tissues histolysis, imaginal progenitor cell development and adult structure formation [13]. These transition events are strictly coordinated by increasing molting hormone (ecdysone) levels and an absence of juvenile hormones (JHs) in last-instar larva. The production and release of ecdysone is regulated by a small secreted neuropeptide, prothoracicotropic hormone (PTTH). PTTH stimulates ecdysone synthesis by regulating the transcription of a series of Halloween genes in the prothoracic gland, including *phantom* (*phm*), *disembodied* (*dib*), and *shadow* (*sad*) [14–18]. Ecdysone is subsequently released into the hemolymph and converted to 20-hydroxyecdysone (20E) by the P450 monooxygenase *shade* (*shd*) in peripheral tissues [19, 20]. The active form of ecdysone, 20E, binds to its nuclear receptor ecdysone receptor (EcR) and forms a heterodimer with the retinoid X receptor ortholog ultraspiracle (USP). EcR/USP binds to DNA and activates the transcription of target genes such as *broad* (*Br*) to amplify ecdysone signaling and direct physiological, morphological, behavioral changes during metamorphosis [16, 21, 22].

The red flour beetle *Tribolium castaneum* (Herbst) (Coleoptera, Tenebrionidae) is an important stored grain pest which avoids light, and all stages of its life cycle develop within the food substrate [23]. It is suspected that the low-UV environment of this beetle makes it highly sensitive to UV [11, 23]. Previous research has shown that UVA irradiation can influence the expression profiles of stress-responsive genes such as *Hsps* and *P450s*. However, it does not cause serious ecological damage to *T*. *castaneum* (for example, to its fecundity or survival) [24]. Nevertheless, little is known about how beetles react to UVB stress. Whether the development, metamorphosis and ecdysteroid metabolism affected by UVB radiation has never been studied. Therefore, in the present study, we performed dose-response analyses of the effects UVB irradiation on the metamorphic process, verified the function of *T*. *castaneum ptth* gene, and examined ecdysteroid pathway gene expression profiles as well as ecdysterone titers of *T*. *castaneum* post UVB irradiation.

## Materials and Methods

### Insects

The beetles were reared on 5% yeast supplemented with whole-wheat flour at 30°C under 50 ± 2% relative humidity in constant darkness. Approximately 200 adults (1–7 days old) laid eggs in flour for one hour, and the eggs were collected and transferred to new diet after separation with a 60-mesh sieve. The last-instar larvae were used in the analysis.

### Measurements of developmental time, larval mortality, pupal size and emergence rates

UVB irradiation was performed at 30°C under 50 ± 2% relative humidity in a dark room. A UVB lamp (UVB8W29T5; Dingguo Changsheng Biotechnology Co. Ltd., Wuhan, Hubei, China) providing irradiance of 800 μw/cm^2^ was used as a light source. Thirty isolated naked last-instar larvae (20 days from oviposition) were placed in a 7 cm-diameter Petri dish without a cover and exposed to radiation at 5 kJ/m^2^ (800 μw/cm^2^ X 625 s), 10 kJ/m^2^ (800 μw/cm^2^ X 1250 s) or 20 kJ/m^2^ (800 μw/cm^2^ X 2500 s). Larvae were subjected to the same procedure without UVB exposure as a control. After irradiation, the larvae were returned to the original diet. The numbers of dead larvae, pupae and emerged adults were recorded every other day. All experiments were repeated three times. The weight and length of the pupae were measured on the second day of pupation.

### Total RNA extraction and *Trcptth* gene cDNA cloning

Total RNA was isolated from the whole bodies of the beetle larvae using TRIzol reagent (Thermo Fisher Scientific Inc., Waltham, MA, USA) and purified using DNase I (Thermo Fisher) according to the manufacturer’s instructions. The cDNA was synthesized using a RevertAid^™^ first strand cDNA synthesis kit (Thermo Fisher). The primers for cloning *Trcptth* gene cDNA were designed against the prothoracicotropic hormone sequence (TC030081) in BeetleBase (http://www.beetlebase.org/?q=home). Full-length sequence was obtained via rapid amplification of cDNA ends (RACE) using the SMARTer^™^ RACE cDNA amplification kit (Takara Biotechnology Co. Ltd., Dalian, Liaoning, China). All primers were detailed in Table 1.

**Table 1:** Primers used in experiments.

Gene	Forward primer 5’–3’	Reverse primer 5’–3’	Efficiency (%)	*R*^2^
**qPCR primers**				
*phm*^a^	AAGAATGTGTGTCGGTGATGAA	TCGTGAGGTTTCGGAGTTAGTG	104.2	0.993
*dib*^a^	ACAGGAAGAGCCACCTCACC	ACCATTCGGGTCCATTTGTT	90.8	0.996
*sad*^a^	GCTAAGAGCCCGCAAATCC	GGTAAAGCCGCAAAGTCTCCT	94.7	0.995
*shd*^a^	GGTCAACGAACAAGGTGAGG	GAGTCGGTCTGCGATGTAGTTT	93.6	0.998
*EcR*^b^	GATGGATGGCGAAGATCAGT	ACTTCGCTGGAACATGCTTT	92.1	0.993
*usp*^b^	GATGCAAGCACAGGATGCTA	CCGACTTTATCCCTCGAACA	90.4	0.991
*Br*^c^	CACAACACTTCTGTCTGCGGTG	CACAGGGTGTTTGCAAGGAG	93.7	0.991
*Kr-h1b*^c^	TGTGACGTTTGCTCGAAGAC	GCACGAGTAGGGCTTTTCAC	91.9	0.993
*Trcptth*	ATGACGAAGTGGATGATCGGTG	GGCCGTGAAGGATGGTAATAAGG	102.6	0.991
*RpS3*^d^	CACCCCAACTCGCACGGA	GCAATGGCACACAGACCCCT	97.8	0.998
**RACE primers**				
*Trcptth*-Clone	TTCACGAAACCGATGGATA	ATGCGACTGTTACTGTGATTGT		
*Trcptth*-3RACEouter	TTAGGACACCAGTATTACCCAAGA			
*Trcptth*-3RACEinner	CCGAAACCCTGAAATAAGACCC			
*Trcptth*-5RACEouter	GGATCTTTCTGTTTCAAAACGCGGACT			
*Trcptth*-5RACEinner	GGCCGTGAAGGATGGTAATAAGGTGAC			
**RNAi primers**				
*dsTrcptth*	TAATACGACTCACTATAGGGACGAGGACATGACGTACCAA	TAATACGACTCACTATAGGGTGAGGGTCTTATTTCAGGGT		
*dsEGFP*	TAATACGACTCACTATAGGGACGTAAACGGCCACAAGTTC	TAATACGACTCACTATAGGGAAGTCGTGCTGCTTCATGTG		

^a^*phm*, *dib*, *sad* and shd primers were described by Hentze et al. (2013).

^b^*EcR* and *usp* primers were described by Parthasarathy et al. (2010).

^c^*Br* and *Kr-h1b* primers were described by Parthasarathy et al. (2008).

^d^*RpS3* primers were described by Sang et al. (2015).

### Bioinformatic and phylogenetic analysis of *Trcptth* gene

The sequences of previously reported PTTH protein were retrieved from the NCBI GenBank database (http://www.ncbi.nlm.nih.gov/) and used for comparative analysis. Multiple sequence alignments were conducted with Clustal W. to determine the conserved domains. A phylogenetic tree was inferred from the amino acid sequence using the maximum likelihood method (model WAG) in MEGA 5.2.2 and tested with a bootstrap of 1,000 replicates to ascertain the reliability of a given branch pattern in the maximum likelihood tree. Signal peptide was predicted with SignalP-4.1Server (http://www.cbs.dtu.dk/services/SignalP/).

### *Trcptth* gene dsRNA preparation and RNA interference

For dsRNA synthesis, the PCR products were used as templates which primers against specific regions of *Trcptth* and *EGFP* (the *EGFP* fragment was amplified from Pub-nls-EGFP vector provided by Dr. Hong-yu Zhang, Huazhong Agricultural University, which was originally from Dr. Handler, USDA) with minimal T7 polymerase promoter sequences at their 5’-ends (Table 1). PCR conditions were 98°C 30 s, followed by 40 cycles at 98°C for 10 s, at 63°C for 30 s and 72°C for 30 s, and 72°C for 5 min. The dsRNA was synthesized with the T7 polymerase using the HiScribe^™^ T7 *in vitro* transcription kit (New England Biolabs Inc., Ipswich, MA, USA) according to the manufacturer’s protocol with 1 μg of purified template. To remove the template DNA, 2 μl of DNase I (Thermo Fisher) was used and the reaction mixture was incubated at 37°C for 15 min. Then dsRNA was purified by isopropanol and sodium acetate and adjusted to 2 μg/μl by 0.1 mM potassium phosphate buffer (containing 5 mM KCl, pH 7.0) for microinjection [25]. Subsequently, the larvae were aligned on a plastic board covered with double-sticky tape and each larva was injected with 400–500 ng of dsRNA until the abdomen was full and the body was visibly extend. After 24 hours, the insects were used to analyze interference efficiency. In addition, the numbers of pupae were recorded. The non-injected, ds*EGFP* and potassium phosphate buffer (saline) -injected larvae were performed as controls.

### Reverse transcriptase quantitative PCR analysis

Last-instar larvae (20 days from oviposition) were randomly selected for exposure to 5, 10, or 20 kJ/m^2^ of UVB, then allowed to recover in a dark environment for 2 hours for a short-term response analysis of gene expression. In addition, larvae from the same stage were exposed to 20 kJ/m^2^ UVB then allowed to recover for 48, 96 or 144 hours in a dark environment, provided with an appropriate diet, for a long-term response analysis. Controls were subjected to the same procedure without UVB irradiation. After the recovery period, the whole bodies of the beetle larvae were immediately frozen in liquid nitrogen and stored at -80°C. Then RNA extraction was performed with TRIzol reagent (Thermo Fisher). The purity and concentration of the RNA were estimated with agarose gel electrophoresis and BioPhotometer Plus (Eppendorf, Hamburg, Germany; 1.9 < OD_260/280_ < 2.0). One microgram of RNA was reverse transcribed to cDNA using the PrimeScript RT reagent kit with gDNA eraser (Takara) following the manufacturer’s instructions. RT-qPCR primers were designed using Primer 3 software [26] and sequences were validated against the *T*. *castaneum* genome database (http://www.ncbi.nlm.nih.gov/genome/guide/beetle/index.html). The gene *RpS3* was used as reference gene in all experiments and amplification efficiencies of all primers were between 90.4–104.2% (Table 1) [27]. RT-qPCR was conducted with the SYBR premix ExTaq (Takara) in a final reaction mixture volume of 20 μl. The reactions were performed with a Bio-RAD iQ2 thermocycler (Bio-Rad Laboratories Inc., Hercules, CA, USA) with the following conditions: initial denaturation at 95°C for 30 s, followed by 40 cycles at 95°C for 20 s and at 60°C for 45 s. The relative expression was calculated using the 2^ΔΔCt^ method [28].

### Determination of ecdysterone levels

For determination of the ecdysterone titer, larvae were collected every six hours from 18 to 22-day-old (from oviposition, which were the last-instar larvae), then washed thoroughly in sterile water, dried on filter paper, and immediately frozen with liquid nitrogen and stored at -80°C. The treatment group larvae were irradiated with 20 kJ/m^2^ of UVB radiation at the 44^th^ hour (18.25 day was defined as 0 hour), whereas the controls were not irradiated. Twenty insects were homogenized in 400 μl of ice-cold 0.65% normal saline and were centrifuged at 3,000 rpm for 10 min at 4°C. The supernatants, 20 μl, were added to 30 μl of sample diluent for testing with the insect ecdysterone ELISA kit (Dingguo Changsheng), according to the manufacturer’s instruction [29, 30]. The optical densities (O.D.) at 450 nm were read using a microplate spectrophotometer (xMark^™^; Bio-Rad) within 15 min. The ecdysterone quantification was calculated with standard curves (standard substances were provided with the kit), and values were presented as picogram per insect (pg/insect). Control and treatment samples of each time point before UVB treatment, were pooled to analyze. Each experiment was repeated three times.

### Statistical analysis

All data analyses were performed using SPSS 18.0 software (SPSS Inc., Chicago, IL, USA). Percentage data were arcsine-square root transformed. Cumulative pupation was analyzed by using repeated measure ANOVA. Emergence rates were analyzed by using Pearson’s χ2-test. Mortality, pupal length, mass and gene expression were analyzed by one-way ANOVA or Student's t-test. Tukey’s multiple range test was used for pairwise comparison of the difference between treatments for mean separation. A *p*-value of less than 0.05 was considered to be statistically significant.

## Results

### Effects of UVB radiation on *T*. *castaneum* metamorphosis, larval mortality, pupal size and emergence rates

**Fig 1:**
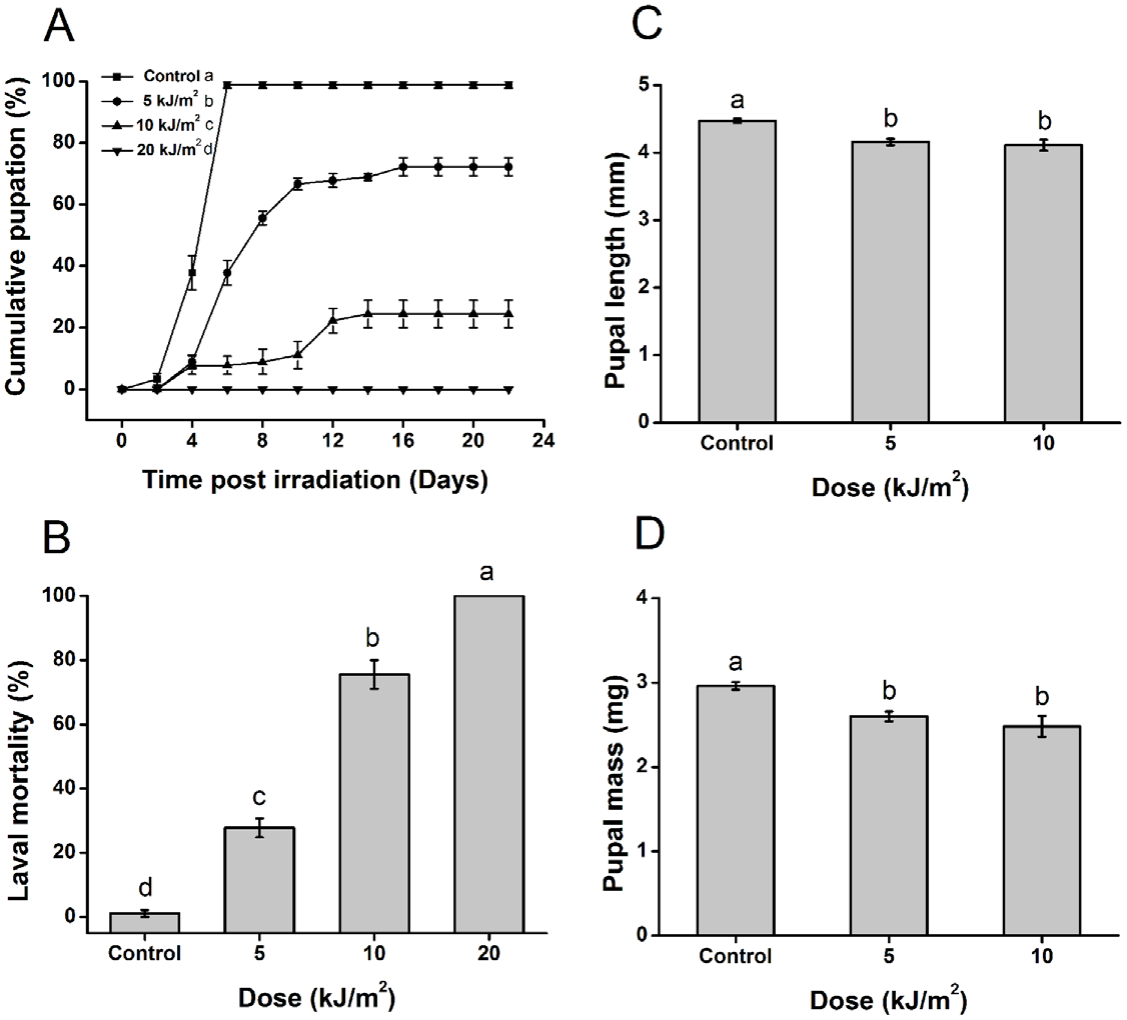
UVB radiation delays *T*. *castaneum* pupation, increases larval mortality, reduces pupal length and mass. Values represent means ± SE. (A) Cumulative pupation of larvae post-UVB irradiation; (B) Larval mortality at 22 days post-irradiation; (C) Pupal length post-irradiation; (D) Pupal mass post-irradiation (Repeated measure ANOVA, (A) F = 277.907, df = 3, 8, *p* < 0.001; One-way ANOVA, (B) F = 228.904, df = 3, 11, *p* < 0.001; (C) F = 20.129, df = 2,137, *p* < 0.001; (D) F = 15.923, df = 2,137, *p* < 0.001). Values or graphic symbols followed by the different letter are significantly different.

To evaluate the effects of UVB radiation on the metamorphosis of *T*. *castaneum*, different doses of UVB radiation were applied to last-instar larvae (20 days from oviposition), and pupation was analyzed. With an increase of the UVB dose from 0 (control) to 20 kJ/m^2^, cumulative pupation was significantly reduced. The insect failed to pupate under the 20 kJ/m^2^ treatment (F = 277.907, df = 3, 8, *p* < 0.001) (Fig 1A). Additionally, metamorphosis was delayed. Six days after UVB treatment, all of larvae in the control group developed into pupae, with the exception of a small number of deaths. However, in the 5 kJ/m^2^ and 10 kJ/m^2^ UVB treatment groups, the insects pupated for 16 and 14 days, respectively. Although a few larvae remained alive after these time points, they died at 22 days post-irradiation. In the 20 kJ/m^2^ UVB treatment groups, larvae could not pupate, but a few of them could survive until 18 days post-irradiation (data not shown). It was found that under an increasing UVB dose, larval mortality increased (F = 228.904, df = 3, 11, *p* < 0.001) (Fig 1B). Although some larvae developed into pupae after 5 kJ/m^2^ or10 kJ/m^2^ exposure, the length and mass of the pupae were significantly reduced (length, F = 20.129, df = 2,137, *p* < 0.001; mass, F = 15.923, df = 2,137, *p* < 0.001) (Fig 1C and 1D). Moreover, the emergence rates were decreased to 71.2% at 5 kJ/m^2^ (χ^2^ = 16.804, *p* < 0.001) and 61.1% at 10 kJ/m^2^ (χ^2^ = 20.247, *p* < 0.001), while that in the control was 97.1%.

### Sequence analysis of *Trcptth* gene in *T*. *castaneum*

**Fig 2:**
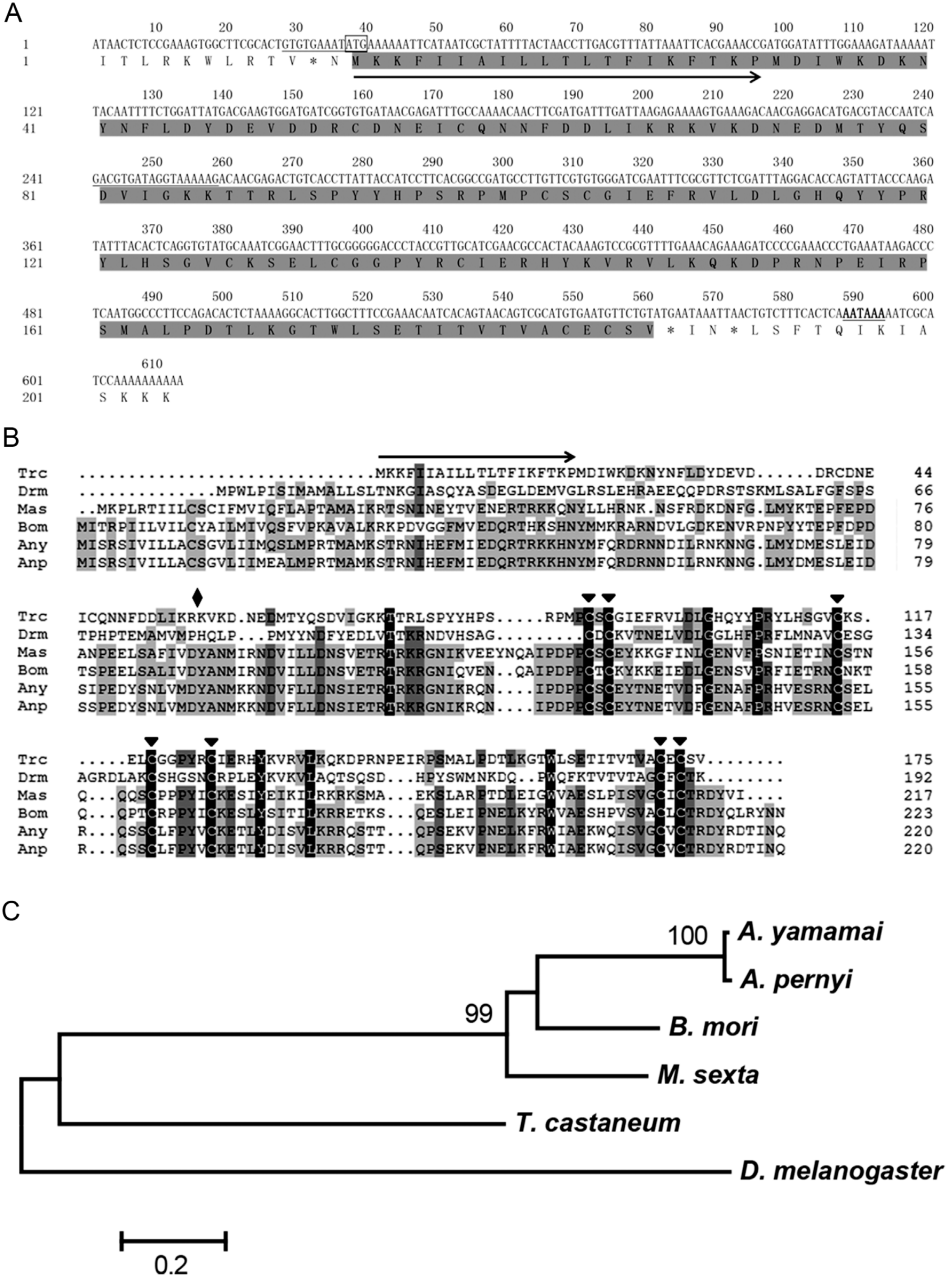
Sequence, alignment and phylogenetic analysis of prothoracicotropic hormone (PTTH) in *T*. *castaneum*. (A) Structure of cDNA sequence and deduced amino acid sequences of TrcPTTH. The start codon (ATG) is boxed; the stop codon (TGA) is marked asterisk; two sequence gaps are underlined; the putative polyadenylation signal (AATAAA) is underlined and bold; putative signal peptide is arrowlined; (B) Sequence alignment analysis of TrcPTTH was compare with PTTHs from *Drosophila melanogaster* (Drm), *Manduca sexta* (Mas), *Bombyx mori* (Bom), *Antheraea pernyi* (Anp) and *Antheraea yamamai* (Any). Solid diamonds indicates the putative cleavage sites that release mature PTTH, and seven filled triangles indicate seven conserved cysteines; Arrowline indicates putative signal peptide. Conserved amino acid residues are indicated as white letters in a black background; similar residues are indicated as black letters in a grey background; non-conserved residues are indicated as black letters in a white background; gaps, indicated as broken lines, are inserted to optimize the alignment. The numbers of amino acid residues are designated arbitrarily for sequence alignment and are not their exact positions in the full sequences; (C) Phylogenetic analysis PTTHs of *T*. *castaneum*, *D*. *melanogaster*, *M*. *sexta*, *B*. *mori*, *A*. *pernyi* and *A*. *yamamai*. The maximum likelihood tree was inferred from amino acid sequences using the WAG model in MEGA 5.2.2. The percentage values at the nodes in the tree indicate bootstrap support in 1,000 replicates. Bootstrap values lower than 70% are not shown.

To address whether UVB radiation influence on metamorphosis of *T*. *castaneum* were related to expression of *ptth*, the exact sequence and function of *Trcptth* gene were verified. The cDNA full length of *Trcptth* gene was 613 bp, which contained a 528 bp open reading frame (ORF), a 36-bp 5’ untranslated region (UTR), and a 49-bp 3’ UTR (GenBank accession number: KM925014). There were two different sites between our sequence and the beetle database (TC030081) marked with underlining (Fig 2A). At the first different site (nt 28–39), there were a stop codon (nt 31–33) and a start codon (nt 37–39) in our sequence (Fig 2A). The deduced TrcPTTH has 175 amino acids with a calculated molecular mass of 20.5167 kDa and an isoelectric point of 8.31. A multiple sequence alignment showed that TrcPTTH is 37%, 37%, 33%, 28%, and 30% identical to the PTTHs from *Antheraea pernyi* Guérin-Méneville (GenBank accession number AAB05259), *Antheraea yamamai* Guérin-Méneville (AAR23822), *Bombyx mori* L. (NP_001037349), *Manduca sexta* L.(AAG14368), and *D*. *melanogaster* Meigen (NP_608537), respectively. The deduced mature TrcPTTH is 37%, 37%, 33%, 28%, and 30% identical to the PTTHs from *A*. *pernyi*, *A*. *yamamai*, *B*. *mori*, *M*. *sexta*, and *D*. *melanogaster*, respectively. The protein consists of a putative signal peptide (20 aa), a precursor domain (58 aa) and a PTTH mature peptide (97 aa) (Fig 2A and 2B). Seven cysteine residues were present within the mature PTTH at conserved locations, as in other known PTTHs (Fig 2B), which are essential for generating full biological activity [31]. The phylogenetic tree inferred based on TrcPTTH and related five PTTHs above showed that. TrcPTTH was well segregated from which of moths and *D*. *melanogaster* (Fig 2C).

### *Trcptth* RNAi influences metamorphosis

**Fig 3:**
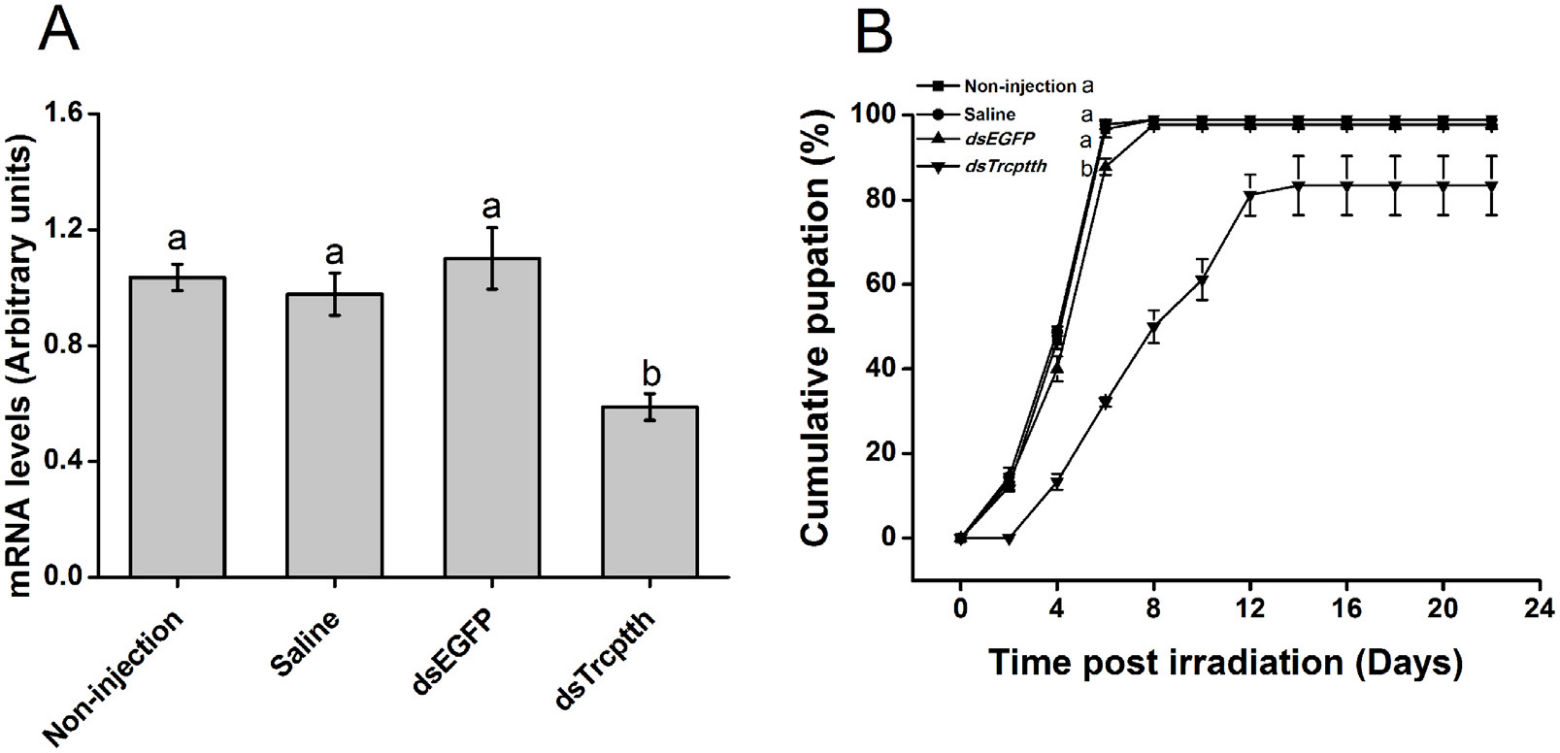
The mRNA levels of *Trcptth* and cumulative pupation in ds*Trcptth* injected and control insects. Values represent means ± SE. (A) mRNA levels of *Trcptth* at 24 hours after dsRNA injection; (B) Cumulative pupation of larvae following dsRNA injection ((A) One-way ANOVA, F = 10.168, df = 3, 11, *p* < 0.01; (B) Repeated measure ANOVA, F = 231.923, df = 1, 8, *p* < 0.001). Values or graphic symbols followed by a different letter are significantly different.

To determine the function of the *Trcptth* gene *in vivo*, dsRNAs were injected into 20-day-old larvae. The mRNA levels of *Trcptth* were reduced by approximately 1.7-fold in insects injected with ds*Trcptth* compared with controls (F = 10.168, df = 3, 11, *p* < 0.01) (Fig 3A). The pupation of ds*Trcptth* injected insects was significantly delayed (6 days) compared with non-injected and saline and *EGFP* dsRNA-injected control insects (F = 231.923, df = 1, 8, *p* < 0.001) (Fig 3B). However, pupal mass was not changed (data not shown).

### Effects of UVB radiation on expression of *Trcptth* and ecdysteroid biosynthesis genes

**Fig 4:**
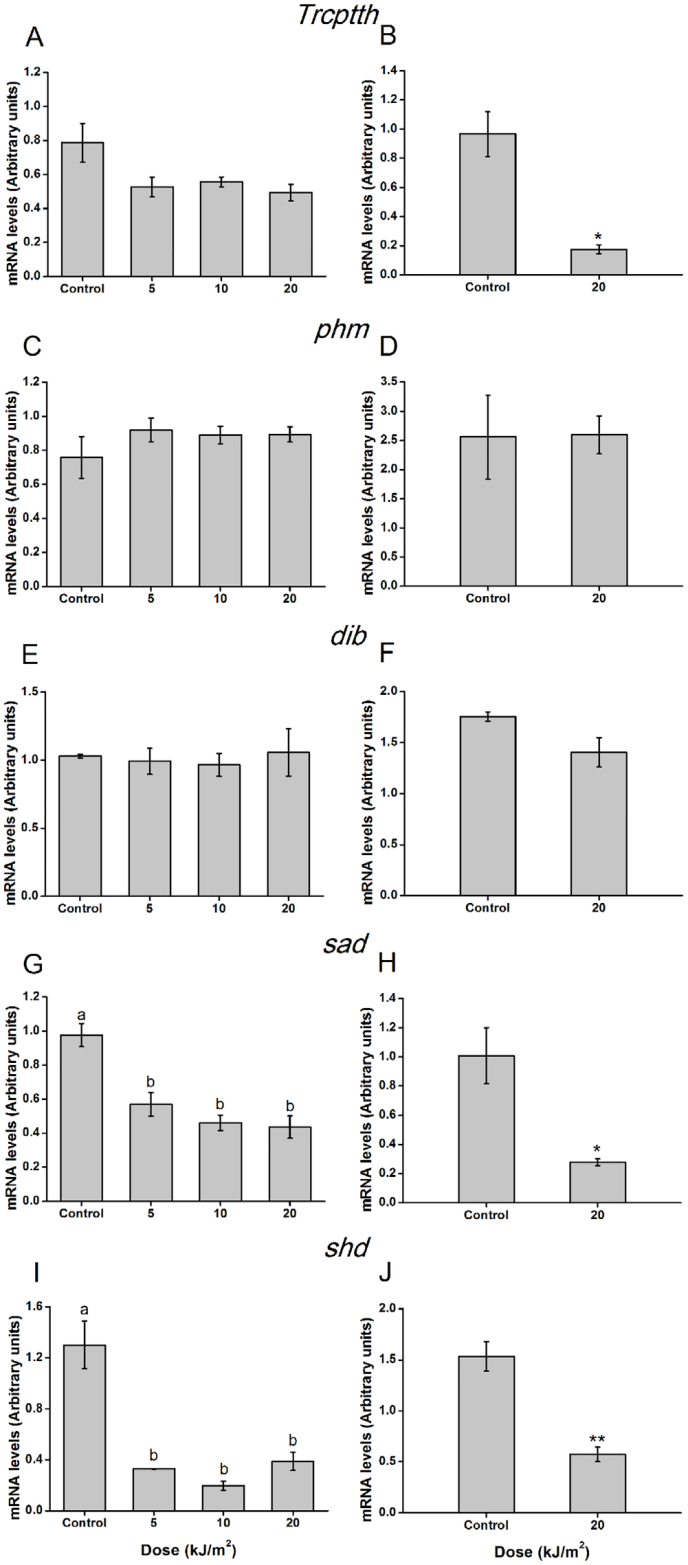
Effects of UVB radiation on expression levels of *Trcptth* and ecdysteroid biosynthesis genes of *T*. *castaneum* larvae at 2 and 48 hours post-UVB irradiation. Values represent means ± SE. (A) *Trcptth*; (C) *phm*; (E) *dib*; (G) *sad*; (I) *shd* expression patterns at 2 hours post different dose exposure; (B) *ptth*; (D) *phm*; (F) *dib*; (H) *sad*; (J) *shd* expression patterns at 48 hours post 20 kJ/m^2^ exposure. (One-way ANOVA, (A) F = 2.837, df = 3, 11, *p* = 0.106; (C) F = 0.883, df = 3,11, *p* = 0.490; (E) F = 0.137, df = 3,11, *p* = 0.935; (G) F = 15.892, df = 3,11, *p* < 0.01; (I) F = 24.668, df = 3,11, *p* < 0.001; Student's t-test, (B) t = 5.049, df = 2.156, *p* < 0.05; (D) t = 0.047, df = 4, *p* = 0.965; (F) t = 2.317, df = 4, *p* = 0.081; (H) t = 3.786, df = 4, *p* < 0.05; (J). t = 5.933, df = 4, *p* < 0.01). The mRNA level was determined by quantitative RT-qPCR. Values followed by a different letter are significantly different. One and two asterisks denote statistically significant difference compared to control (*p* < 0.05 and *p* < 0.01, respectively).

To assess the effects of UVB irradiation on ecdysteroid synthesis signaling, RT-qPCR was used to examine UVB-induced changes in the expression of *Trcptth* and Halloween genes including *phm*, *dib*, and *sad*, which catalyze ecdysone synthesis, as well as *shd*, which converts ecdysone to 20E. Two hours after exposure to different doses of UVB, the expression level of *Trcptth* was not significantly changed (F = 2.837, df = 3, 11, *p* = 0.106) (Fig 4A). However, 48 hours post-exposure to 20 kJ/m^2^, the expression of *Trcptth* declined significantly (t = 5.049, df = 2.156, *p* < 0.05) (Fig 4B). The expression levels of *phm* and *dib* were unaffected at 2 and 48 hours after treatment with any dose of UVB radiation (2 hours: *phm*, F = 0.883, df = 3,11, *p* = 0.490; *dib*, F = 0.137, df = 3,11, *p* = 0.935; 48 hours: *phm*, t = 0.047, df = 4, *p* = 0.965; *dib*, t = 2.317, df = 4, *p* = 0.081) (Fig 4C, 4E, 4D and 4F). In contrast, *sad* and *shd* were dramatically repressed at 2 hours after exposure to all of the tested doses (*sad*, F = 15.892, df = 3,11, *p* < 0.01; *shd*, F = 24.668, df = 3,11, *p* < 0.001) (Fig 4G and 4I). Forty-eight hours later, *sad* and *shd* were observed to be consistently down-regulated (*sad*, t = 3.786, df = 4, *p* < 0.05; *shd*, t = 5.933, df = 4, *p* < 0.01) (Fig 4H and 4J). Notably, at 96 and 144 hours post-exposure, the expression levels of *sad* and *shd* were still very low (S1 Fig).

### Effects of UVB radiation on ecdysterone titer

**Fig 5:**
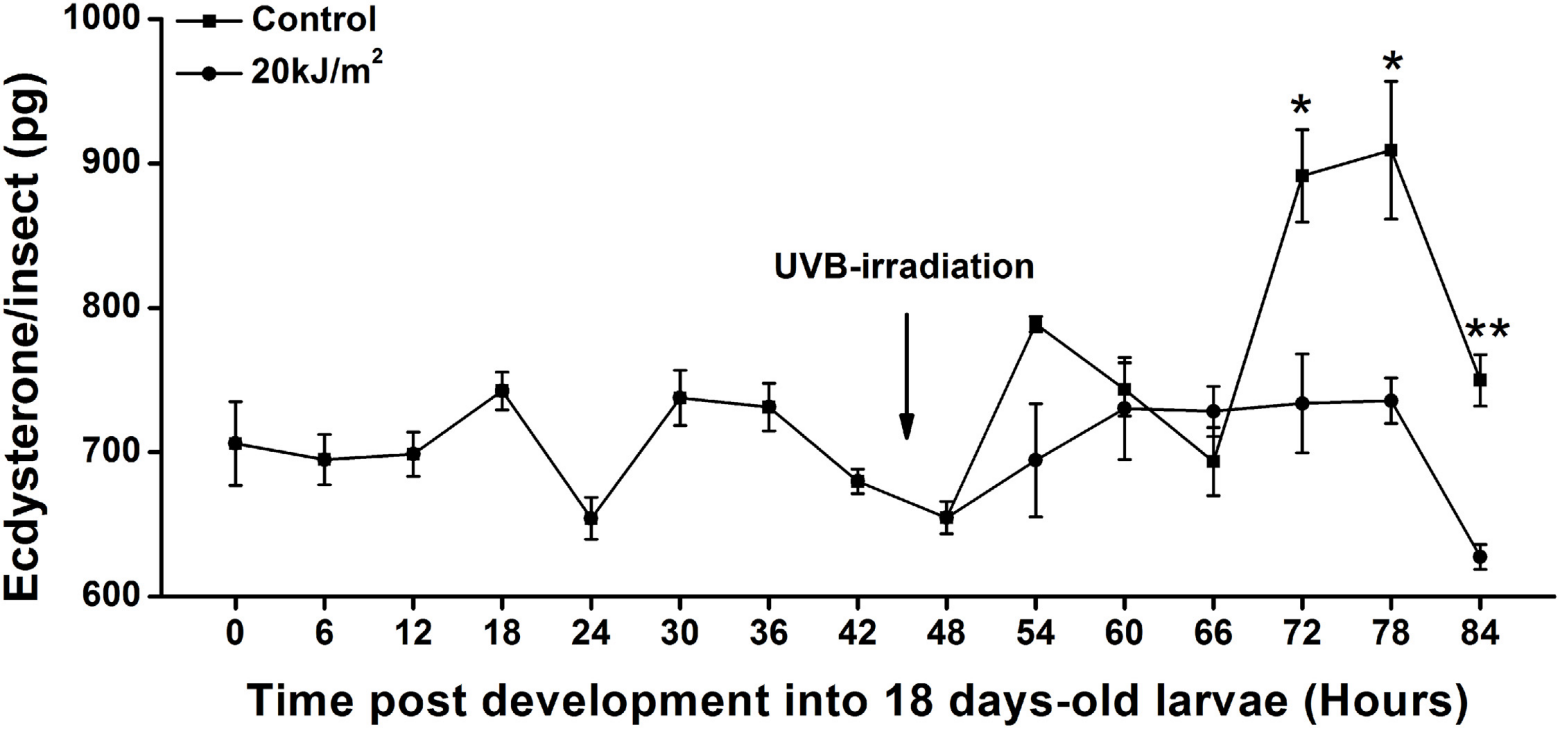
Estimation of ecdysterone levels in the final-instar larval stages of *T*. *castaneum*. Values represent means ± SE. Ecdysterone levels of insects were analyzed every 6 hours during the final-instar larval stage (0–84 hours). The larvae at 0-hour point indicates eggs developed 18.25 days. UVB treatment was performed at the 44^th^ hour. Data were analyzed at each time point by using Student's t-test, 54^th^ hour, t = 2.384, df = 2.073, *p* = 0.136; 60^th^ hour, t = 0.328, df = 4, *p* = 0.760; 66^th^ hour, t = 1.192, df = 4, *p* = 0.299;72^nd^ hour, t = 3.364, df = 4, *p* < 0.05; 78^th^ hour, t = 3.457, df = 4, *p* < 0.05; 84^th^ hour, t = 6.182 df = 4, *p* < 0.01. One and two asterisks denote statistically significant difference compared to control (*p* < 0.05 and *p* < 0.01, respectively).

To compare ecdysterone titers in irradiated and non-irradiated larvae, ecdysterone levels were examined every six hours from 18 to 22- day-old larvae (Fig 5). The ecdysterone levels of non-irradiated larvae were low during the first 48 hours, and increased at 54^th^ and 72^nd^ hours. However, in UVB-irradiated larvae, the ecdysterone generation showed no peaks, and decreased significantly at 72^nd^, 78^th^ and 84^th^ hours compared with controls (72^nd^ hour, t = 3.364, df = 4, *p* < 0.05; 78^th^ hour, t = 3.457, df = 4, *p* < 0.05; 84^th^ hour, t = 6.182 df = 4, *p* < 0.01) (Fig 5).

### Effects of UVB radiation on expression of 20E- and JH-responsive genes

**Fig 6:**
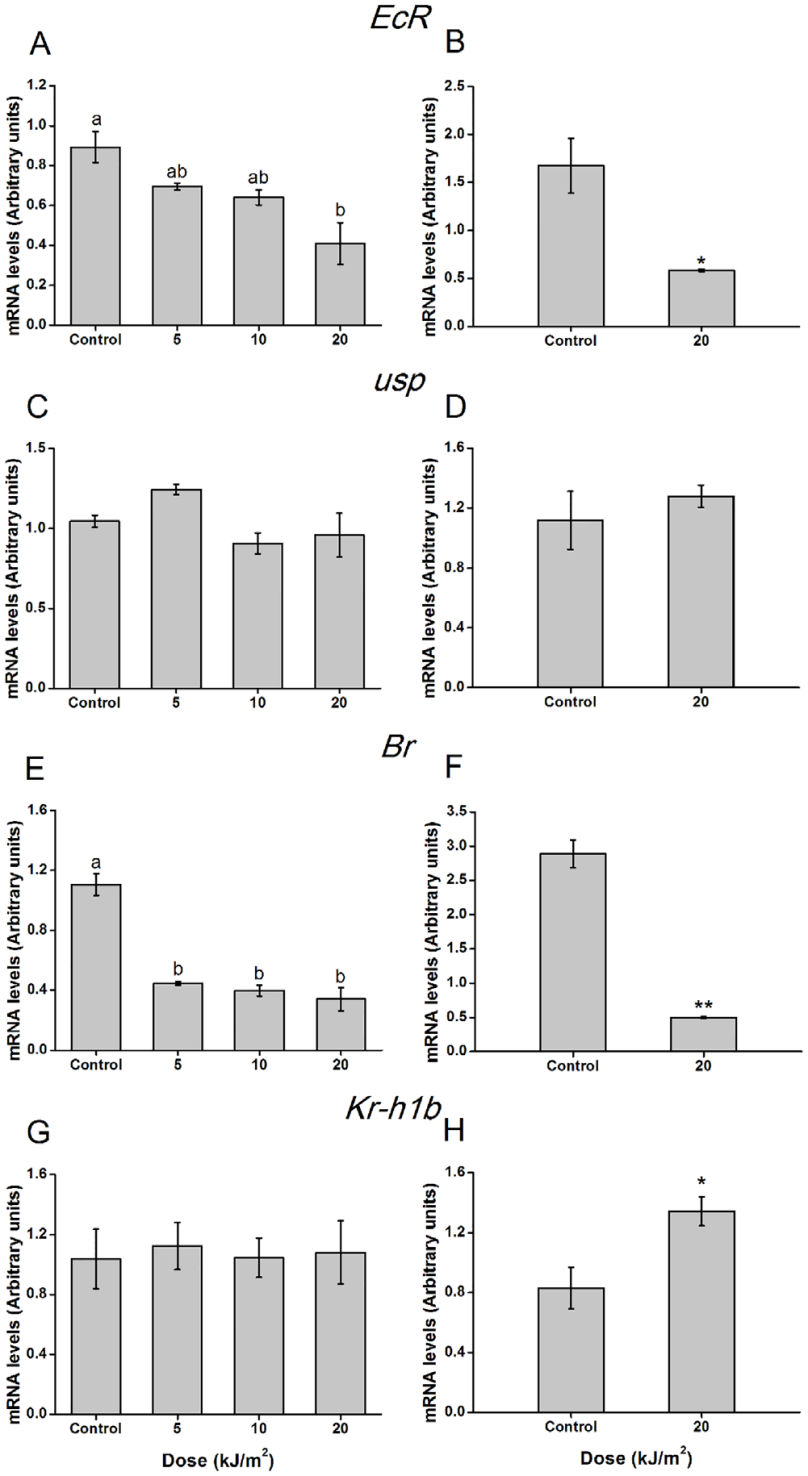
Effects of UVB radiation on expression levels of 20E- and JH-responsive genes of *T*. *castaneum* larvae at 2 and 48 hours post-UVB irradiation. Values represent means ± SE. (A) *EcR*; (C) *usp*; (E) *Br*; (G) *Kr-h1b* expression patterns at 2 hours post different dose exposure; (B) *EcR*; (D) *usp*; (F) *Br*; (H) *Kr-h1b* expression patterns at 48 hours post 20 kJ/m^2^ exposure (One-way ANOVA, (A) F = 8.552, df = 3,11, *p* < 0.01; (C) F = 3.372, df = 3,11, *p* = 0.075; (E) F = 40.488, df = 3,11, *p* < 0.001; (G) F = 0.049, df = 3,11, *p* = 0.985; Student's t-test, (B) t = 3.797, df = 4, *p* < 0.05; (D) t = 0.774, df = 4, *p* = 0.482; (F) t = 11.720, df = 4, *p* < 0.001; (H) t = 3.055, df = 4, *p* < 0.05). The mRNA level was determined by RT-qPCR. Values followed by a different letter are significantly different; One and two asterisks denote statistically significant difference compared to control (*p* <0.05 and *p* < 0.01, respectively).

To determine the effects of UVB radiation on the expression of 20E- and JH-responsive genes, the expression levels of *EcR*, *usp*, *Br* and *Kr-h1b* were analyzed. The expression level of *EcR* was reduced by the 20 kJ/m^2^ treatment (F = 8.552, df = 3, 11, *p* < 0.01), and the expression level of *Br* was reduced under all doses at 2 hours post-irradiation (F = 40.488, df = 3, 11, *p* < 0.001) (Fig 6A and 6E). At 48 hours after the 20 kJ/m^2^ treatment, the expression profiles of *EcR* and *Br* were consistent, showing very low levels (*EcR*, t = 3.797, df = 4, *p* < 0.05; *Br*, t = 11.720, df = 4, *p* < 0.001) (Fig 6B and 6F). Even after 96 and 144 hours, the expression of these genes remianed repressed (S2 Fig). Nevertheless, at 2 and 48 hours after exposure to any dose of UVB radiation, the expression of *usp* was not affected (F = 3.372, df = 3, 11, *p* = 0.075; t = 0.774, df = 4, *p* = 0.482) (Fig 5C and 5D). Notably, the expression of *Kr-h1b* increased significantly at 48 hours after 20 kJ/m^2^ irradiation, whereas it was not altered at 2 hours post-exposure (F = 0.049, df = 3,11, *p* = 0.985; t = 3.055, df = 4, *p* < 0.05) (Fig 6G and 6H).

## Discussion

UVB radiation can impact the larval-pupal metamorphosis and survival of *T*. *castaneum*. UVB-induced death of insects has been reported in previous studies, which is consistent with our results [11, 12]. In the present study, the development and metamorphosis of last-instar larvae of *T*. *castaneum* were prevented or delayed by UVB irradiation (Fig 1A). UVB-induced disruption of amphibian development was first detected in *R*. *pipiens* Schreber, whose embryos showed developmental delay after 3 days of UVB exposure from a quartz mercury-lamp. This study demonstrated that UVB could not only cause outright mortality, but could also have sublethal effects (impediments to development) [32]. Croteau summarized 12 studies on amphibian species showing that UVB radiation emitted from sunlight or an artificial source can induce various changes in amphibian development and metamorphosis, including delays and acceleration [33]. In the insect *M*. *sexta* L., UVB can only delay development of first-instar larvae and does not affect fifth-instar larvae [11]. *T*. *castaneum* is a pest of stored grain, and all stages of its life cycle develop within the food substrate. The low-UV environment of this beetle makes it highly sensitive to UV. Strategies for coping with UVB might have been lost in this species, resulting in obvious UVB-induced effects. Therefore, the observed variation in the influence on development and metamorphosis might be related to the species and developmental stages. UVB induced high mortality in larvae under increasing doses, but the larvae did not die immediately after irradiation (Fig 1B). Indeed, most of the insects survived for quite a long time post-irradiation, surviving for more than 16 days in some groups (data not shown). The UVB-induced damage to organisms is mainly related to DNA lesions. If these lesions overwhelm the DNA repair system, they lead to mutation. Although larvae still lived on post irradiation, mutations accumulate continuously with DNA replication and mitotic, ultimately resulting in abnormal gene expression and physiological disorders [2–4]. It is only when the accumulation of disorders reaches a level that exceeds the repair ability of insects that death will occur. Surviving insects were able to pupate and emerge after low-dose UVB exposure, but their pupal size was significantly reduced (Fig 1C and 1D). Animal body size is determined by the duration of the growth period and the amount of nutrition obtained during that period [34]. UVB delayed metamorphosis and increased the growth period, but the pupae became much smaller, indicating that the insects either reduced their nutrient intake or increased their nutrient consumption, which could be related to a decline in feeding activity or too great an energy input for repair activities. To our knowledge, this is the first report indicating that UVB can influence the metamorphosis of insects.

PTTH is a neurohormone that could stimulate prothoracic glands to produce ecdysteroids and regulate insect metamorphosis [14–18]. Insect neurosecretory neurons that synthesize PTTH can receive many environmental stimuli through receptor systems, including cold, heat, photoperiod, injury, and plant allelic chemicals stimuli [35–37]. As UVB is a common environmental stimulus in nature, we wondered whether the UVB-induced changes in *T*. *castaneum* metamorphosis were related to the expression of *Trcptth* and downstream genes such as *phm*, *dib*, *sad*, and *shd*. However, the function of the *ptth* gene in *T*. *castaneum* had not been experimentally verified prior to the present study [38]. Therefore, we assessed the sequence and function of the *Trcptth* gene of *T*. *castaneum*. The results of phylogenetic analysis showed that TrcPTTH formed a single clade, distinct from other PTTHs from moths and *D*. *melanogaster* (Fig 2C). RNAi results demonstrated that *Trcptth* could control *T*. *castaneum* pupation (Fig 3). It has been reported that tissue damage caused by x-irradiation and cell ablation can induce a delay in development of *Drosophila*, as these injuries produce signals that trigger decreased *ptth* expression [39, 40]. The present study showed that UVB irradiation did not influence *Trcptth* gene expression in the short term (within 2 hours) but did significantly down-regulate *Trcptth* after 48 hours. In contrast, the downstream Halloween genes *sad* and *shd*, which are expressed in the prothoracic glands and in peripheral tissues, were significantly down-regulated at 2 and 48 hours post-irradiation (Fig 4). This funding implied that UVB radiation produces a signal that might exert its action not by decreasing *Trcptth* expression from neurosecretory cells during the rapid response period, but through the synthesis and activation of genes from the prothoracic glands and peripheral tissues. In later stage (48 hours), *Trcptth*, *sad* and *shd* might regulate ecdysone signaling together.

Normally, ecdysteroid levels are low in *T*. *castaneum* after larvae develop to the last instar. In the case of an increase in ecdysteroid levels in last-instar larvae in the absence of JHs, the larvae would be committed to pupation [41]. In the present study, reduction of ecdysteroidogenic gene expression after UVB irradiation could have resulted in a decline of hemolymph ecdysterone titer levels (Figs 4 and 5). When the UVB radiation treatment occurred before the commitment point, the pulse of ecdysteroid was disturbed. Subsequently, the larval-pupal transition was delayed or prevented. Similar results have been reported in *D*. *melanogaster*, where injury to the wing can lead to a global developmental delay associated with down-regulation of ecdysteroidogenic genes and repression of ecdysone signaling components [39].

Activated ecdysone (20E) should bind to the EcR nuclear receptor to initiate the transcription of early response genes, such as *Br*, to complete the metamorphosis of salivary glands, fat bodies, the gut and dorsal flight muscles [41, 42]. In turn, high titers of JH up-regulate Kr-h1 via the Met or Gce receptor, which could repress the expression of *Br* and 20E-induced caspase-dependent programmed cell death during larval molts in *Drosophila* [43]. Therefore, *Br* represents a central component of the action of 20E and JH. In *T*. *castaneum*, RNAi-mediated knock-down of the *Br* gene in the final-instar larval stage derails larval-pupal metamorphosis [41]. In the present study, forty-eight hours after UVB irradiation, the level of *Br* expression was significantly reduced, whereas that of *Kr-h1b* was induced (Fig 6). These findings implied that UVB-treated insects might maintain high JH levels, which would prevent larval molting and metamorphosis. It has been reported that nutritional deprivation, high temperatures and microbial invasion can also elevate JH in *M*. *sexta* L. [44]. Environmental stress-induced changes in insect endocrine signaling are an adaptive physiological strategy. Such events lead to changes in development and cause animals to attempt to restore homeostasis before proceeding with their complex life cycle, in addition to avoiding unfavorable conditions to enhance survival [35, 44]. Therefore, further studies addressing the influence of UVB radiation on JHs should be performed.

In conclusion, this study demonstrated that UVB radiation induced a delay in *T*. *castaneum* metamorphosis, which was related to effects on ecdysteroid pathway regulation. This work will contribute to a better understanding of the impact of UVB on signaling mechanisms in insect metamorphosis.

## Supporting Information

S1 Fig RT-qPCR analysis the expression levels of *Trcptth* and ecdysteroid biosynthesis genes at 2, 48, 96 and 144 hours post-UVB irradiation. (DOCX)

S2 Fig RT-qPCR analysis the expression levels of 20E- and JH-responsive genes at 2, 48, 96 and 144 hours post-UVB irradiation. (DOCX)
