# Supplementary material for: UVB Radiation Delays Tribolium castaneum Metamorphosis by Influencing Ecdysteroid Metabolism
Source: PLoS One. 2016 Mar 17;11(3):e0151831. doi: 10.1371/journal.pone.0151831 (PMC4795627; doi:10.1371/journal.pone.0151831)
Supplement: S2 Fig — (DOCX) [file pone.0151831.s002.docx]

**S2 Fig. RT-qPCR analysis the expression levels of 20E- and JH-responsive genes at 2, 48, 96 and 144 hours post-UVB irradiation.**
